# Cultivating Confident Care: Implementation of a Bitesize Teaching Approach on a Crisis Assessment Unit

**DOI:** 10.1192/bjo.2025.10279

**Published:** 2025-06-20

**Authors:** Andrew Hayward, James Selbie, Laura Shaw

**Affiliations:** 1 Leeds and York Partnership NHS Foundation Trust, Leeds, United Kingdom; 2 The Leeds Teaching Hospitals NHS Trust, Leeds, United Kingdom

## Abstract

**Aims:** Following a transformation in the acute care pathway within Leeds and York Partnership NHS Foundation Trust, a six-bed inpatient unit was established to support service users experiencing complex mental health crises requiring an in-depth assessment period of up to 72 hours. This service redesign highlighted a need for multidisciplinary staff to enhance their confidence in formally assessing a service user’s mental state within this timeframe and managing common medical issues, including those associated with the initiation of psychotropic medication.

To address this, we implemented a structured programme of regular ‘bitesize’ teaching sessions. The primary objective was to improve staff competence in these key areas, while a secondary aim was to embed regular teaching into the workplace culture, fostering continuous professional development within the multidisciplinary team.

**Methods:** A poster was developed to raise awareness of the teaching programme and gather staff input on topic selection. Nine structured 15-minute teaching sessions were delivered by various members of the multidisciplinary team and strategically integrated into regular board round meetings to optimise attendance.

To evaluate the impact of these sessions, attendees were asked to subjectively rate their confidence levels before and after each session using a five-point Likert scale. Additionally, an objective knowledge assessment was conducted, and qualitative feedback was collected. Pre- and post-session Likert scale data were compared and analysed for statistical significance using the Wilcoxon signed-rank test.

**Results:** A total of 71 staff members participated in the nine sessions, with further sessions planned as part of the ongoing programme. Attendees included doctors, nurses, occupational therapists, health support workers and healthcare students, demonstrating broad multidisciplinary engagement.

Both verbal and written feedback were overwhelmingly positive, with recurring themes highlighting the sessions as engaging, accessible, and informative. Across all teaching topics, improvements in both knowledge and confidence levels were consistently observed, with the latter achieving statistical significance. However, challenges in ensuring the regular delivery of sessions were noted, particularly in relation to staff availability due to workload pressures and securing presenters.

**Conclusion:** The findings suggest that the ‘bitesize’ teaching model is an effective and well-received approach to enhancing staff confidence and knowledge in mental state assessment and the management of common medical conditions. The sessions continue to run, and to further support learning, a compendium of educational resources is being compiled for future reference and accessibility.

